# Eco-evolutionary dynamics in a disturbed world: implications for the maintenance of ecological networks

**DOI:** 10.12688/f1000research.15629.1

**Published:** 2019-01-24

**Authors:** Nicolas Loeuille

**Affiliations:** 1 iEES Paris (UMR7618), Sorbonne Université, CNRS, 4 Place Jussieu, 75005 Paris, France

**Keywords:** eco-evolutionary dynamics, evolutionary rescue, species coexistence, ecological networks, phenology

## Abstract

Past management of exploited species and of conservation issues has often ignored the evolutionary dynamics of species. During the 70s and 80s, evolution was mostly considered a slow process that may be safely ignored for most management issues. However, in recent years, examples of fast evolution have accumulated, suggesting that time scales of evolutionary dynamics (variations in genotype frequencies) and of ecological dynamics (variations in species densities) are often largely comparable, so that complex feedbacks commonly exist between the ecological and the evolutionary context (“eco-evolutionary dynamics”). While a first approach is of course to consider the evolution of a given species, in ecological communities, species are interlinked by interaction networks. In the present article, I discuss how species (co)evolution in such a network context may alter our understanding and predictions for species coexistence, given the disturbed world we live in. I review some concepts and examples suggesting that evolution may enhance the robustness of ecological networks and then show that, in many situations, the reverse may also happen, as evolutionary dynamics can harm diversity maintenance in various ways. I particularly focus on how evolution modifies indirect effects in ecological networks, then move to coevolution and discuss how the outcome of coevolution for species coexistence depends on the type of interaction (mutualistic or antagonistic) that is considered. I also review examples of phenotypes that are known to be important for ecological networks and shown to vary rapidly given global changes. Given all these components, evolution produces indirect eco-evolutionary effects within networks that will ultimately influence the optimal management of the current biodiversity crisis.

## Introduction

In spite of the many examples presented by Darwin of fast evolution, especially in the context of artificial selection ^1^, the potential for evolution to affect ecological dynamics has been largely ignored in the prediction and management of species exploitation or species conservation. Until recently, the time scale of evolution was often considered to be so long ^2^ that it could be safely ignored for many applied topics in ecology. While this view dominated during the 70s and the 80s, many results in the past two decades have accumulated, showing that evolution alters ecological dynamics, even on short timescales. The evolution of species directly targeted by humans is easy to perceive. The development of modern medicine has led to the repeated evolution of resistance in bacteria ^3^ and the intensification of agriculture to the fast evolution of pesticide resistance in various pest species ^4^. Such examples may give the false impression that fast evolution matters only for small, short-lived species. On the contrary, other examples clearly point out that such phenomena apply broadly, even for larger species. Large differences in the survival of different heritable phenotypes can, for instance, produce large evolutionary variations, even in one generation. Fast evolution of age and size at maturity in a cod fishery has been observed in just a few years ^5^. This evolution eventually constrained cod recovery when fishing stopped. Evolution of leg morphology in cane toads has allowed this invasive species to propagate increasingly quickly in Australia ^6^. Next to these particular examples, more general analyses show the impact of evolution on the demography of species as well as on the dynamics of their interactions. Reconsidering previously published species, Hairston *et al*. ^7^ showed that the population growth rate of different species from different case studies was affected equally by (ecological) density or environment-dependent effects and by (evolutionary) changes in their phenotypes, suggesting that evolution happens on a time scale that is relevant for ecological dynamics. Classical predator–prey population cycles are similarly affected, with many datasets suggesting that evolution often shapes such cycles ^8– 10^.

Fast evolutionary dynamics should be all the more prevalent in the context of current global changes. Global changes are of political and societal importance because they cause important declines in many species, affecting either their survival or their fecundity. Because these two quantities are the basic fitness components, global changes imply strong selective pressures ^11– 13^ so that fast evolution is expected for any phenotype that would be heritable, variable, and associated with these variations in fecundity and survival. Fast evolution has been repeatedly shown in the case of invasive species, both in alien species ^6, 14, 15^ and in species of the recipient community ^16, 17^. It then largely alters the dynamics of the invasion and its effects on invaded ecosystems. Evolution under climate change has been similarly observed. It modifies species phenologies ^18– 21^ and constrains changes in species distributions ^22– 24^. Evolution in response to overexploitation ^5, 25, 26^ or in response to agricultural management ^27, 28^ has also been extensively documented.

The question of the role of evolution in conservation issues is thus particularly important and increasingly recognized ^29, 30^. Evolution may help the conservation of diversity. For instance, the idea of evolutionary rescue ^31^ proposes that, following a disturbance, if natural selection acts fast enough, it may allow local species adaptation and survival, as the evolving species’ growth rate is restored by evolution. While many instances of evolutionary rescue have been observed in nature ^30, 32^ and the conditions of its occurrence theoretically and experimentally investigated, its general importance for the overall maintenance of diversity is still unknown. Particularly, evolutionary rescue is a concept based on a monospecific approach ^31^ and its impact on the dynamics of the network in which the evolving species is embedded is still largely unknown. However, experimental evidence highlights that such effects do exist. For instance, experimental evolution of plants depending on humidity conditions alters the composition and structure of their microbial communities ^33^, thereby affecting plant–soil feedbacks.

Other works suggest less optimistic impacts of evolution on species diversity. Evolution under frequency-dependent selection (i.e. the fitness of individuals of a particular phenotype depends on whether this phenotype is rare or common in the population) can drive the extinction of the evolving species (evolutionary suicide ^34– 36^). While frequency dependence may sometimes be beneficial from a fitness point of view, current evidence shows that it restricts the applicability of evolutionary rescue ^37^. Evolution can also directly decrease population size (evolutionary deterioration ^38^), thereby increasing the probability of extinction of the species. Evolution of a species can also lead to the loss of another species in the network (evolutionary murder ^39, 40^). Ultimately, the overall effect of species evolution on the maintenance of diversity under global changes will depend on which of these processes (evolutionary rescue, suicide, deterioration, and murder) dominate.

In the present article, I focus on the implications of species (co)evolution within networks, given the context of our disturbed world. I tackle three questions: (1) what are the implications of evolutionary rescue in a network context? (2) Does the effect of evolution on diversity depend on the type of interaction (hence the type of network) that is considered? (3) Are the traits with documented variations linked to global changes important in a network context?

## Evolutionary rescue in the context of ecological networks

**Figure 1.**
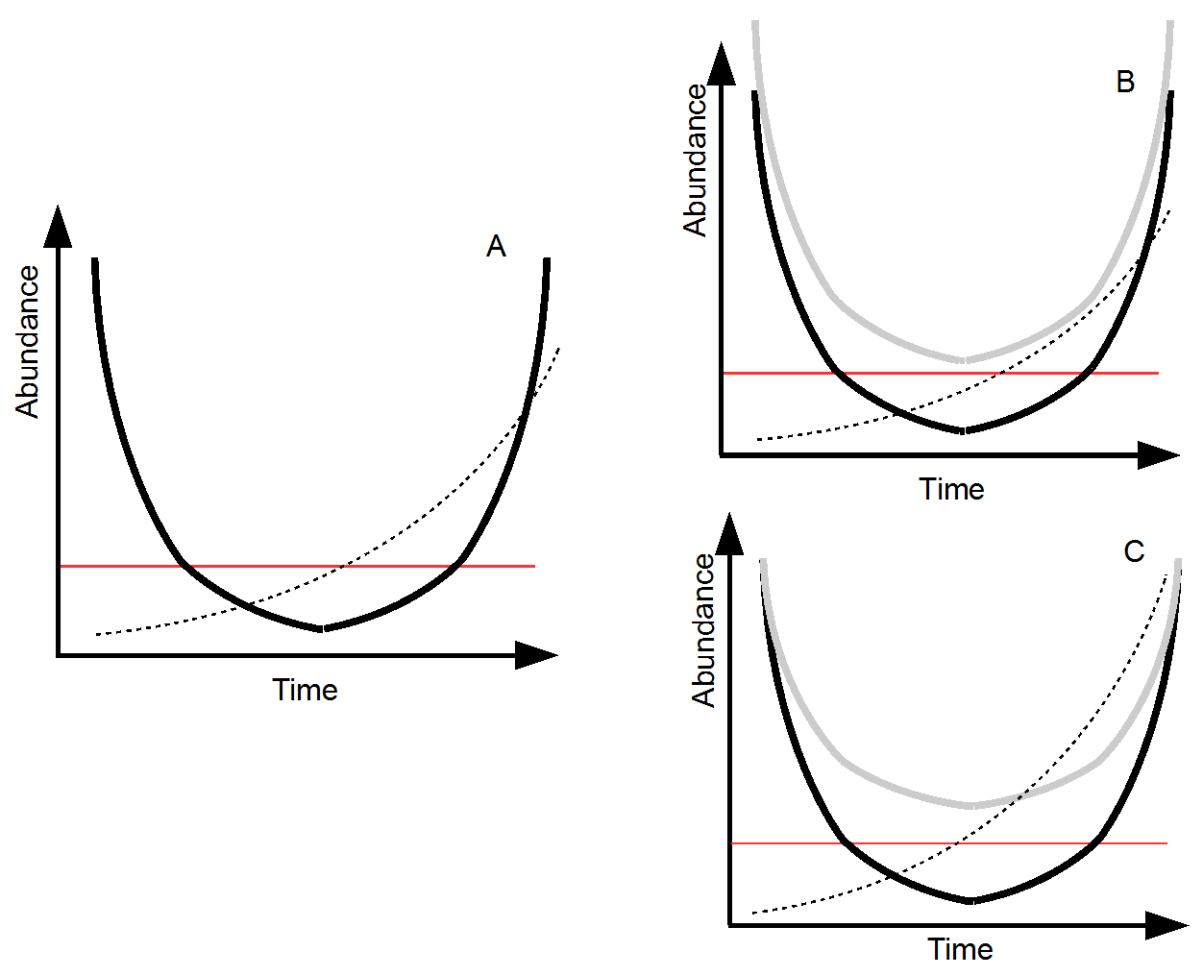
Evolutionary rescue. ( **A**) The black line shows the evolving species’ density. At the start, the species undergoes an alteration in its environment that leads to a negative population growth rate. However, natural selection favors adapted alleles in the population (the dashed line shows the adapted allele frequency). This adaptation increases the species’ growth rate. When this growth rate becomes positive, the species’ density increases again. The red line shows the population under which extinction is likely (e.g. due to demographic stochasticity). The longer the species spends under this threshold, the larger the probability of extinction. ( **B**) Evolutionary rescue depends on population size. In this panel, the only difference between the black and the gray species is initial population size. Evolutionary rescue is more likely for the gray species, as its larger initial population leaves more time for evolution to act before the threshold is reached. ( **C**) Evolutionary rescue depends on genetic variability. In this panel, the two species differ only in their genetic variability. The gray species initially has a larger genetic variability. This allows a faster evolutionary response, thereby facilitating rescue. Adapted from 30, 31.

Given current changes, the role of species adaptation is hotly debated. While some studies claim that niche conservatism should prevail ^41, 42^, others have pointed out that rapid evolutionary adaptation plays an important role in the maintenance of diversity ^18, 21, 43– 45^. Under evolutionary rescue, a species may adapt following a selection process born from a change in its environment and survive because of this adaptation. While such an outcome offers important hope given the current biodiversity crisis, it likely applies to a restricted set of species ( Figure 1). Reviews on the conditions of application of evolutionary rescue have been published elsewhere (e.g. 30). Species with large populations are more likely to survive through evolutionary rescue ( Figure 1). Large populations offer more time for evolution to act before the species abundance is dangerously low. Also, larger abundances often offer more genetic variability (e.g. more reproductive events, hence the possibility of transmission of more *de novo* mutations) ^46, 47^. Similarly, species that have faster life-cycles are more likely to be saved by evolutionary rescue, as the numerous reproductive events allow the accumulation of new mutations. A higher genetic variability promotes evolutionary rescue ( Figure 1). Because most species of conservation concern do not have high abundances and often have slow time cycles, evolutionary rescue is unlikely to save these species.

**Figure 2.**
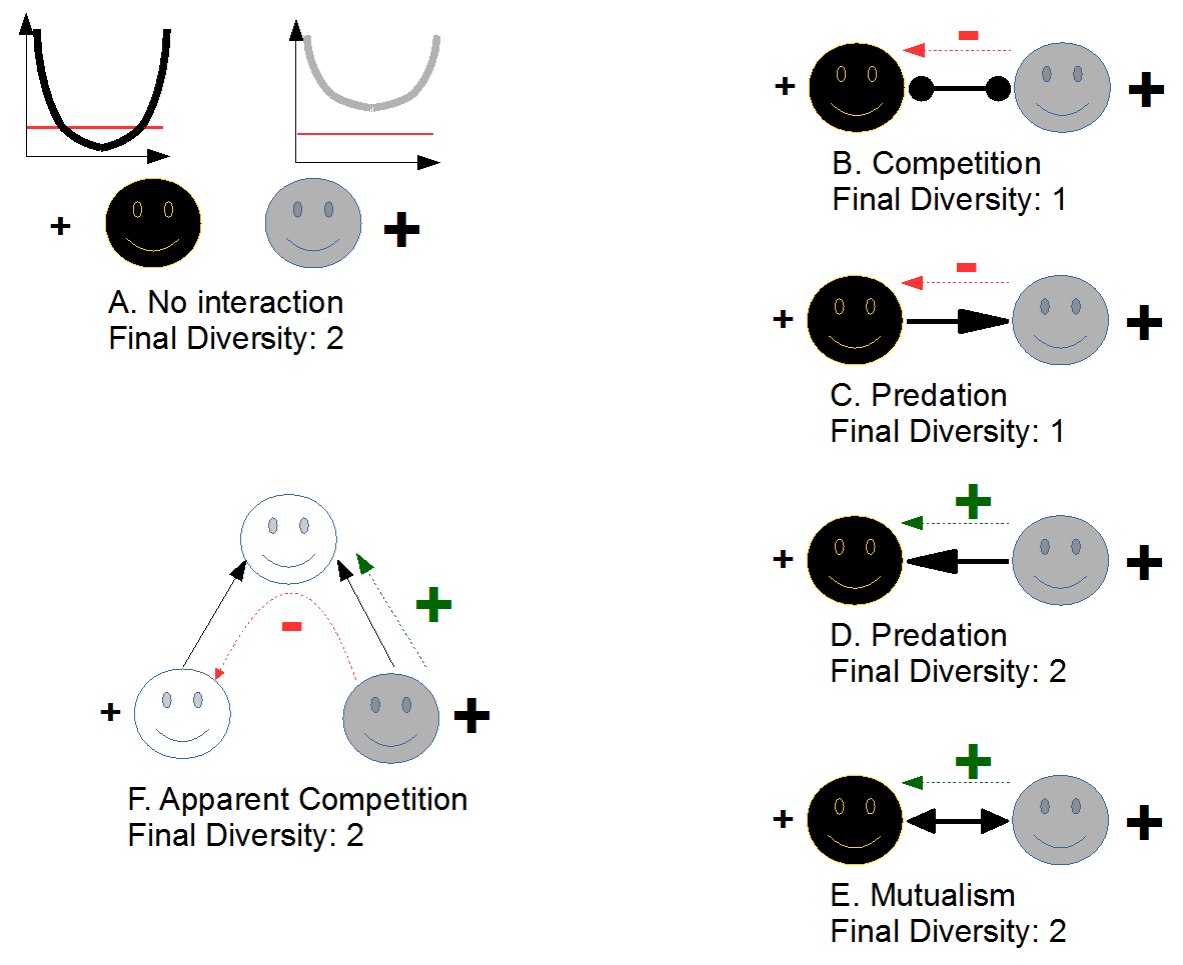
Evolutionary rescue, considering ecological interactions. ( **A**) Starting from the classical, monospecific view of evolutionary rescue, direct effects of evolution are positive for the maintenance of diversity (black +). Note, however, that evolutionary rescue is more efficient for species A (gray) than for species B (black) (larger + sign). ( **B**) When the two species compete, evolutionary rescue favors one of the two species, possibly leading to the loss of the other species (evolutionary murder). ( **C**) Similarly, when species A is a predator of species B, evolutionary rescue may decrease diversity by increasing top-down effects. However, efficient evolutionary rescue in species A may actually help species B, for instance by increasing bottom-up effects ( **D**) or when the two species have mutualistic interactions ( **E**). Effects of evolutionary rescue may propagate further. If two prey species share a predator and one species has a very efficient evolutionary rescue, this helps to maintain the predator species (through bottom-up effect) but may lead to the evolutionary murder of the other prey species, as apparent competition is increased. In all panels, ecological interactions are in solid arrows, direct effects of evolutionary rescue on diversity maintenance are shown by a black +, and indirect effects of evolutionary rescue on diversity are depicted using green or red signs.

I do not want to focus on these already-reviewed aspects ^30^; instead, I would rather question the implications of evolutionary rescue outside of the monospecific framework in which it has been grounded to bring it into a network context. Imagine that we have two species in a network undergoing a disturbance in their abiotic environment (e.g. a temperature change). Following this disturbance, imagine that the two species adapt to the change through natural selection (e.g. through modifications of their thermal niche). I want to stress here that the trait I consider is not directly selected for by species interactions but rather selected by the (abiotic) environment. Finally, imagine that one of the two species undergoes an efficient evolutionary rescue process (hereafter species A), while the other one does not (hereafter species B), for instance because of asymmetries in abundances or in initial genetic variabilities. Even though both species undergo evolutionary rescue and both would likely survive if one were to consider species separately ( Figure 2A), the network context may alter this prediction. If the species are in competition ( Figure 2B), species B will likely be killed (evolutionary murder ^39, 40, 48, 49^) because the efficient evolutionary rescue in species A leads to a competitive asymmetry between the two species. Similarly, if we imagine that species A is now a predator of species B ( Figure 2C), its efficient evolutionary rescue enhances the decline of species B, likely driving it to extinction. Species B indeed suffers simultaneously from the outside disturbance (evolutionary rescue being hardly efficient for species B) and from the ecological context (more predators, as species A has an efficient evolutionary rescue). Following the extinction of species B, species A may similarly go extinct if it is a specialist or may survive if it consumes other prey in the network. In the latter case, the efficient evolutionary rescue in species A will directly impact all of its prey in the food web. Now consider the reverse case: species B is the predator ( Figure 2D). The ecological context now enhances the positive effect of evolutionary rescue on coexistence. The very efficient evolutionary rescue in prey species A creates bottom-up effects on its predator (species B). Species B may then hardly suffer from the external change. Similar positive effects are expected if the two species have mutualistic interactions ( Figure 2E). Effects extend beyond pairwise interactions. If one considers two prey species that share a predator (a classical “apparent competition” module ^50^), efficient evolutionary rescue in species A will help to maintain its predator but may be detrimental to the maintenance of the other prey species ( Figure 2F). In conclusion, while evolutionary rescue may help the focal species to survive in deteriorated environments (at least under some conditions), its effect on diversity as a whole, accounting for community structure, is not likely to be systematically positive. Rather, it will lead to some important surprises, as evolutionary rescue modifies indirect effects happening within the ecological network.

Some empirical observations can be linked to these ideas. For instance, the evolution of resistance in agricultural pests incurs large losses in terms of agricultural productivity ^30, 51^. This clearly highlights how evolutionary rescue in consumer species reduces the abundance and productivity of lower trophic levels. Considering competition, several evolutionary models suggest that while species evolution can help the survival of some species through adaptation, as well as the colonization of new ranges, such winners are compensated by the extinctions of many other species that suffer from increased competition ^52, 53^. Observations suggest that the maintenance of mutualistic interactions is also affected by such rescue processes. In the case of coral bleaching, evolutionary variations are likely to be highly important to determine the resilience of coral reefs ^54, 55^. Rapid adaptation is more likely to come from evolution in the symbionts, as they have larger population sizes and faster generations ^54^. Such work suggests that rescue of one of the mutualistic partners helps the maintenance of the whole system, as proposed above. Note that because evolutionary rescue is more likely to happen in (small) species that have short generations and large populations, the cascading effects I introduce here are likely larger when such species have a dominant role in the network’s structure and functioning. Immediate candidates include pathogens, whose abundance and impact on ecological network structure is now well documented ^56^.

## An example: co-evolution of species phenology in plant–herbivore and plant–pollinator systems

As illustrated by Figure 2, how evolution and ecology interact and affect the maintenance of species diversity depends on the type of interaction that is considered (antagonism [competition or predation] versus mutualism). While many studies remain focused on either trophic networks or mutualistic networks, an increasing number of researchers are interested in understanding how mixing different types of interactions affects the stability and diversity of ecological networks ^57– 60^. In an evolutionary context, it has been shown that in a complex ecosystem where several interactions coexist, evolution does not systematically enhance the stability of the network ^61^. Evolution is more stabilizing when one considers the trophic part of the interaction network ^62, 63^, while evolution of the mutualistic interaction is more often destabilizing ^61^. Given present disturbances, it is urgent to develop a more integrative understanding of how ecological networks function, considering the different interaction types they contain.

**Figure 3.**
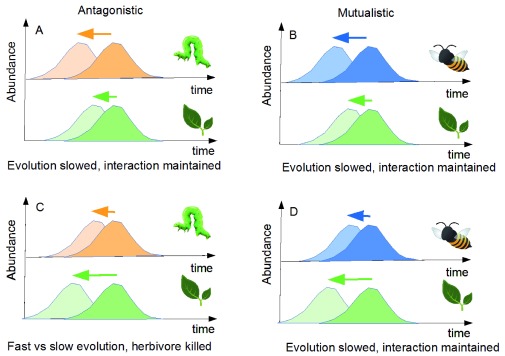
Coevolution of plant–interactor phenologies under different scenarios. Scenarios differ in interaction type (antagonistic on the left [ **A** and **C**] and mutualistic on the right [ **B** and **D**]) and in the species with the higher evolutionary potential (e.g. genetic variability) to shift its phenology in response to climate change (top row: interactor potential higher; bottom: plant potential higher). In each panel, the initial phenology is shown in dark. Possible new phenologies given the evolutionary potential are shown in light. Arrows show the magnitude of the potential shift. A possible outcome for the evolution is proposed below each panel.

Particularly, expected evolutionary feedbacks differ depending on the type of ecological interaction ^57^. To discuss this, I use the evolution of phenologies as a working example ( Figure 3). I consider eco-evolutionary dynamics of plant–herbivore interactions ( Figure 3A and 3C) and of plant–pollinator interactions ( Figure 3B and 3D) to contrast mutualistic and antagonistic interactions. I focus on phenology for several reasons. Changes in phenologies are one of the most prevalent consequences of climatic changes ^64^. Also, previous studies suggest that species vastly differ in their ability to shift ^65, 66^, with important consequences for the maintenance of interspecific interactions in ecological networks ^67– 69^. For instance, a recent review on 15 years in Texas showed that amphibians have shifted their phenologies in different ways, increasing competitive interactions in these communities ^70^. In each case, I assume that the species evolve toward earlier phenologies, for instance because of climatic changes. One of the species (either the plant or its interactor) can evolve a phenological change fast while potential evolution is slower in the other species, for instance because its genetic variability is reduced. I also consider that the overlap of the two phenologies is a proxy of the intensity of the ecological interaction. The different situations are shown in Figure 3.

In Figure 3A, I assume that the herbivore population responds more easily so that its phenological shift is facilitated. Potential evolution for the plant is, on the contrary, assumed to be limited. Given these components, herbivores that emerge early will be resource limited, having little plant to consume. As a result, in such a situation, although the herbivore could in principle exhibit evolutionary rescue (as it is able to evolve fast), it will likely not because its evolution is constrained by the low evolutionary potential of its resource. If this evolution is too slow given current changes, the diversity may not be maintained. Now turn to the reverse scenario ( Figure 3C), in which the plant has a high evolutionary potential while the herbivore cannot shift easily. The plant is now released from some of the top-down effects and its population may grow. This may in turn increase its evolutionary potential (e.g. because of the accumulation of new mutations), further facilitating the rescue process. Therefore, ecological release may speed up plant evolution, accelerating evolutionary rescue. The herbivore, on the contrary, is a victim of two distinct forces. On the evolutionary side, it has a low potential, so that evolutionary rescue is unlikely. On the ecological side, its resource population does shift, so that it is left with little resource. Such forces interact to promote the extinction of the herbivore.

I now turn briefly to the right part of the figure ( Figure 3B and 3D) that shows mutualistic interactions. Regardless of the situation, the species whose evolutionary potential is higher will have no mutualistic partner when shifting its phenology. Assuming the mutualistic interaction has a large impact on fitness, early emerging individuals will be counterselected, and the realized phenological shift will actually be close to the one observed for the slower species. Therefore, I expect that in the case of these mutualistic interactions, the evolutionary response will be close to the potential response of the slower species. This suggests that evolutionary inertia may commonly happen for such interactions. This inertia limits the efficiency of evolutionary rescue and may play a part in the observed decline of pollinators and associated plants observed in different places ^71– 73^. Also, this idea is in line with other studies pointing out the evolutionary vulnerability of mutualisms given current changes ^12^. While previous work suggests that mutualistic networks, involving intrinsically strong positive feedbacks, are prone to sudden collapse ^74^, the present analysis suggests that they may also be less likely to evolutionarily adapt to external disturbances.

Superficially, it may seem that results from Figure 2E and Figure 3B and 3D are in contradiction, as the first points out how mutualism may be beneficial for evolutionary rescue while the other argues the contrary. The two figures actually show different processes. Figure 2 relies on an analysis of density-dependent effects. Indeed, when mutualism enhances densities, it may help evolutionary rescue. Figure 3 relies on the distribution of this density in times (phenologies). Possible mismatch may then limit the rescue process. In a mutualistic context, it is therefore important to understand which of these two processes (density-dependent effect versus mismatch effect) will dominate to properly analyze the fragility of the interaction.

Of course, other scenarios are possible. Analysis of Figure 3 relies on a pairwise interaction, therefore implicitly assuming that both species are specialists (or at the very least that the particular interaction plays an important role in the fitness of both species). For more generalist species, phenological shifts can in fact lead to changes in interaction partners. Such interaction switches have been observed in several instances ^12, 55, 69^. This does not preclude the possibility of negative evolutionary effects on diversity maintenance. Such a rewiring creates new indirect effects in the ecological network ^75^ that may be positive or negative (see Figure 2 and related text). While the effects of evolution on biodiversity in the context of ecological networks require further theoretical developments (but see 76, 77), we already know that some of the traits that largely drive ecological dynamics within these networks are currently varying fast under current changes.

Also, the analysis of Figure 3 relies on evolutionary variations in phenology. While variations in phenology have been widely observed ^64^, the role of plasticity, of the evolution of plasticity, and of genetic changes in such variations is not always clear and likely varies depending on species and on the ecological context. However, several studies have underlined that evolution can clearly be an important part of such shifts. For instance, variations in egg-laying date in great tits are largely explained by either genetic variations ^19^ or evolution of plasticity ^18^. Such a role of evolution in spawning dates has also been pointed out for amphibian species ^21^. Similarly, the timing of bird migration has recently shifted, and part of these shifts is linked to evolutionary changes ^78^.

## Effects of current changes on phenotypes strongly impacting ecological networks

Many studies document the variations of different phenotypic traits under global changes. While the role of evolution versus plasticity is not always clear in these studies, several of these variations are widespread, consistent, and sustained, suggesting a directional selective process whose influence on ecological networks can be important. While a complete list of these phenotypes is beyond the scope of this article, I here discuss a few phenotypic traits satisfying two conditions: (1) documented, consistent variations given current changes and (2) documented impact on the structure or functioning of ecological networks.

Body size, for instance, has large effects on many aspects of the ecological dynamics of species, affecting not only life history traits ^79^ but also the occurrence and intensity of ecological interactions. Predator–prey interactions are largely affected by the distribution of body sizes ^80, 81^, predators being larger than their prey by a given ratio ^82, 83^. Differences in body size also affect competitive interactions ^84^. Therefore, changes in the body size of different species in an ecological network likely impact its structure and functioning, as suggested by empirical data ^85^ and theoretical models ^48, 86– 89^. One of the key theoretical frameworks to understand the implications of body size for ecology is the metabolic theory of ecology ^90^. It also allows some predictions on how increasing temperature (e.g. through climate change) may affect the selection of body sizes. Consistent with these predictions, many empirical data show that current changes lead to decreasing body sizes within natural ecosystems. Evidence is particularly strong for aquatic systems ^91, 92^, where smaller body sizes are selected at different organizational levels, within species and among species ^91^. Selection of smaller body sizes is so prevalent in the empirical literature that it has been proposed as a general law of climate changes ^93^. This leads to two important questions regarding ecological networks. First, what will be the implications of such phenotypic changes for the structure and functioning of these systems? If the various species change body sizes at different rates, modifications in interaction partners are likely. Interaction strengths will also likely be modified, affecting the stability of future networks ^94^. Second, how does the ecological network act as a selective agent on species body sizes? As body size affects ecological interactions, the ecological network may also constrain future body size variations in addition to or in interaction with climatic changes. Selection toward smaller body sizes may be accelerated or dampened. Body size dynamics may also depend on the position of the species within the network. For instance, recent theoretical results suggest that larger body size variations happen at higher trophic levels ^77^.

I now get back to phenological changes. Phenology directly affects the occurrence of interactions within the network by the simple fact that for two species to interact, it is necessary that they co-occur (i.e. their phenologies match). I have already explained some of the evolutionary consequences of changes in phenology. I would like to turn to the consequences of such phenological changes for the ecological network. A first consequence is that shifts in phenology, if constrained in different ways between different groups (for instance between plants and their pollinators), may lead to changes in interaction partners. An immediate consequence is that interaction patterns are altered, and network structures and energy pathways will likely be modified. Also, partners are of different quality, which has important implications for the evolution of such networks. In an interesting experiment published recently, Gervasi and Schiestl ^68^ manipulated the pollinators of different *Brassica rapa* plants, with one pollinator treatment (bumblebees) offering a better service than the other (hoverflies). They showed that in just a few generations, the plants pollinated by hoverflies evolved a decreased investment in advertising traits (shorter plants, modified volatile compounds) and evolved toward more autonomous self-pollination. This study clearly shows possible consequences of partner switches in pollination networks due to phenological changes. Other reproductive modes can be selected and the mutualistic interaction undermined. This is also consistent with other work suggesting that mutualisms can be evolutionarily threatened by current changes owing to partner switches ^12^.

Changes in phenologies in a key interaction also potentially change the functioning of the whole system. Deacy *et al.* ^69^ showed that, because of climate change, in Alaska, the phenologies of red elderberry ( *Sambucus racemosa*) and of sockeye salmon ( *Oncorhynchus nerka*) migration have progressively converged. As a result, Kodiak brown bears ( *Ursus arctos middendorffi*) have relaxed their predation on salmon, as they prefer to eat the elderberries. Because in normal years bear predation is the major source of salmon mortality, and because salmon mortality is a huge part of the nutrient cycling in such ecosystems ^95^, the phenological shift described by Deacy *et al.* likely affects the whole ecosystem’s functioning by changing the way nutrients are spatially distributed.

Other traits certainly deserve investigation. For instance, stoichiometric ratios are important constraints for interspecific interactions and ecosystem functioning ^96, 97^. Because nitrogen deposition and modifications of nutrient cycles by humans are important current changes ^98^, the distribution of available nutrients presently changes in major ways, possibly affecting the selection of stoichiometric ratios in many organisms. Such variations may constrain the transmission of energy within ecological networks, affecting their functioning and stability ^99^. Global changes may also affect the selection on plant chemistry. Recent data show that the number of freezing days impacts the frequency of various thyme phenotypes that differ in their chemistry ^100^. Because plant metabolites play an important role in plant–herbivore interactions ^101^, plant–pollinator interactions, or in both ^102– 105^, such modifications may ultimately affect the structure of multiple interaction networks.

Understanding such eco-evolutionary aspects requires the development of theoretical models allowing a relevant complexity (i.e. multiple species to study the diversity issues) and of relevant phenotypes and trade-offs. While the current developments in community evolution models ^48, 49^ offer proper tools to tackle these issues, such complex questions need time and investigation, which is at odds with the urgency of the situation.
